# A feasibility study of the clinical effectiveness and cost-effectiveness of individual cognitive behavioral therapy for panic disorder in a Japanese clinical setting: an uncontrolled pilot study

**DOI:** 10.1186/s13104-016-2262-5

**Published:** 2016-10-07

**Authors:** Yoichi Seki, Shinobu Nagata, Takayuki Shibuya, Naoki Yoshinaga, Mizue Yokoo, Hanae Ibuki, Noriko Minamitani, Muga Kusunoki, Yasushi Inada, Nobuko Kawasoe, Soichiro Adachi, Kensuke Yoshimura, Michiko Nakazato, Masaomi Iyo, Akiko Nakagawa, Eiji Shimizu

**Affiliations:** 1 United Graduate School of Child Development, Osaka University, Kanazawa University, Hamamatsu University School of Medicine, Chiba University and University of Fukui, Suita, Japan; 2 Research Center for Child Mental Development, Chiba University Graduate School of Medicine, 1-8-1 Inohana, Chuo-ku, Chiba-shi, Chiba, 260-8670 Japan; 3 Department of Cognitive Behavioral Physiology, Chiba University Graduate School of Medicine, Chiba, Japan; 4 Organization for Promotion of Tenure Track, University of Miyazaki, Miyazaki, Japan; 5 Inada Clinic, Osaka, Japan; 6 Clinic Adachi, Gifu, Japan; 7 Department of Psychiatry, Graduate School of Medicine, Chiba University, Chiba, Japan

**Keywords:** Cognitive behavioral therapy, Panic disorder, Japanese, QALY, Quality of life

## Abstract

**Background:**

In Japan, cognitive behavioral therapy (CBT) for panic disorder (PD) is not well established. Therefore, a feasibility study of the clinical effectiveness and cost-effectiveness of CBT for PD in a Japanese clinical setting is urgently required. This was a pilot uncontrolled trial and the intervention consisted of a 16-week CBT program. The primary outcome was Panic Disorder Severity Scale (PDSS) scores. Quality of life was assessed using the EuroQol’s EQ-5D questionnaire. Assessments were conducted at baseline, 8 weeks, and at the end of the study. Fifteen subjects completed outcome measures at all assessment points.

**Results:**

At post-CBT, the mean reduction in PDSS scores from baseline was −6.6 (95 % CI 3.80 to −9.40, p < 0.001) with a Cohen’s d = 1.77 (95 % CI 0.88–2.55). Ten (66.7 %) participants achieved a 40 % or greater reduction in PDSS. By calculating areas under the curve for EQ-5D index changes, we estimated that patients gained a minimum of 0.102 QALYs per 1 year due to the CBT.

**Conclusions:**

This study demonstrated that individual CBT for PD may be useful in Japanese clinical settings but further randomized control trials are needed.

*Trial registration*: UMIN-CTR UMIN000022693 (retrospectively registered)

## Background

Panic disorder (PD) is an anxiety disorder characterized by recurring panic attacks [1]. PD is one of the most prevalent psychiatric disorders in developed and developing countries [2], and its prevalence and incidence rates are very similar across the globe [3]. In Japan, the prevalence of PD is 0.8 % [4]. PD is often comorbid with other psychological disorders (as with many anxiety and depressive disorders), and is associated with functional disability (e.g., social and occupational impairment [2]), low health-related quality of life, and economic burden [5, 6].

Both pharmacotherapy and psychotherapy have been recommended as first-line treatments for PD [7]. Cognitive behavioral therapy (CBT) may be more effective than pharmacotherapy, while combining pharmacotherapy with CBT is superior to the use of antidepressants alone [8]. Furthermore, CBT is more cost-effective for treating PD compared to the use of serotonin re-uptake inhibitors (SSRIs) only [9, 10].

Notably, randomized controlled trials in Western countries have consistently indicated that individual CBT alone is effective for treating PD [11–13]. Furthermore, individually administered CBT appears to be more effective than group therapy [13]. However, in Japan, CBT’s effectiveness for PD has not yet been well established. Therefore, a feasibility study of individual CBT for PD in Japanese clinical settings is urgently required. A feasibility study would clarify whether CBT can achieve favorable treatment outcomes in Japanese PD patients, and whether it is sufficiently cost-effective.

The first purpose of this uncontrolled trial is to clarify the clinical effectiveness of an individual CBT program for PD in Japanese clinical settings. As pointed out by Kaczkurkin and Foa [14], exposure and cognitive therapy are two of the most commonly used CBT methods used to treat anxiety disorders. In contrast, our CBT program for PD is based on cognitive therapy that utilizes the Clark et al. [15] model for PD and the Clark and Wells [16] model for social anxiety disorder. Furthermore, it includes behavioral experiments, as with interoceptive and agoraphobic exposure.

In terms of cost-effectiveness, CBT and CBT combined with an SSRI are considered more cost-effective for treating PD as compared to an SSRI only [17]. An effective indicator of cost-effectiveness used in the past is the quality-adjusted life year (QALY), which combines the outcomes of duration and quality of life in the assessment of medical interventions [18]. Thus, the second purpose of this study was to estimate the number of QALYs gained via our CBT for PD in Japanese clinical settings.

## Methods

### Study design

This study was an uncontrolled and unblinded clinical trial. Because this study was the first trial employing an individual CBT intervention for PD in Japan, we believed an uncontrolled design examining the baseline predictors to be appropriate [19]. Patients were recruited and screened for a diagnosis of PD via an interview before undergoing the CBT intervention. Patients received the CBT intervention for 16 weeks, and assessments were conducted before the first session (at week 0; pre-CBT), after the eighth session (week 8; mid-CBT), and after the final session (week 16; post-CBT). This study protocol was approved by the Ethics Committee of the Chiba University Graduate School of Medicine (Reference number: 1710) and was registered in the national UMIN Clinical Trials registry (ID: UMIN000022693).

### Participants

This study was conducted at three clinics: the outpatient clinic at Chiba University Hospital, Inada Clinic, and Clinic Adachi. Participants were recruited through clinical referrals and web-based advertisements between April 2014 and March 2015. Written informed consent was obtained from all patients before any assessments were made. Criteria for inclusion in this study were a primary diagnosis of PD according to DSM-5 criteria, being between 18 and 65 years of age, and having at least moderately severe PD (according to a Panic Disorder Severity Scale [PDSS] score ≥8; [20]). Comorbid diagnoses were permitted if they were clearly secondary (i.e., the PD symptoms were both the most severe and the most impairing). The exclusion criteria were having psychosis, pervasive developmental disorders/mental retardation, a currently high risk of suicide, substance abuse or dependence in the past 12 months, or antisocial personality disorder. All patients were evaluated by a psychiatrist using the MINI International Neuropsychiatric Interview [21, 22]. Treatment history was confirmed by a therapist and chart review.

### Intervention

The individual CBT intervention was conducted in 16 weekly 50-min sessions. We developed the CBT program for PD to focus on changing catastrophic misinterpretations of bodily sensations, as per the Clark et al. [15] model. We also applied several concepts from the Clark and Wells model for social anxiety disorder [16], because in two recent studies of ours on the effectiveness of CBT for social anxiety disorder—by a single arm trial [23] and a randomized controlled trial [24, 25]—we found such concepts to be effective not only for social anxiety disorder, but also for PD, which are both highly common anxiety disorders. Specifically, we added the concepts of the detrimental effects of safety behaviors, attentional bias modification (attentional shift training), behavioral experiments including interoceptive exposure (systematic exposure to body sensations), imagery and memory rescripting, and reconsideration of worry/rumination to strengthen anticipatory anxiety. The main treatment steps were as follows:

- Development of an individualized version of the cognitive-behavioral model of PD;
- Conducting role-play-based behavioral experiments with and without safety behaviors;
- Restructuring catastrophic self-imagery induced by bodily sensations or catastrophic misinterpretations of bodily sensations [15];
- Practicing external focus and the shifting of attention;
- Behavioral experiments to test negative catastrophic beliefs [26];
- Rescripting early memories linked to negative images in panic situations;
- Modifying problematic pre- and post-event processing;
- Discussing the difference between self-beliefs and other people’s beliefs (reflected in survey results);
- Dealing with the remaining assumptions (schema work); and
- Preventing relapse;

Furthermore, we assigned homework after every session; this was meant to help patients test in daily life their beliefs about each treatment theme that they had identified collaboratively with the therapist.

### Quality control

The CBT was delivered by 9 therapists (7 clinical psychologists and 2 psychiatrists) who were experienced in delivering CBT for PD. To confirm therapists’ adherence to the protocol and assist with the planning of future sessions for each treatment, all of the therapists attended weekly group supervision sessions with other therapists and with a senior supervisor (ES). The senior supervisor also checked the quality of the CBT delivered by therapists using the cognitive therapy scale-revised [27].

### Outcomes

The primary outcome measure was the self-reported severity of PD, as measured by the PDSS [20]. The self-report form of the PDSS [28] measures the severity of PD on a 5-point Likert-type scale ranging from 0 (not severe) to 4 (severe); as such, higher scores indicate more severe PD. This scale was adapted from the original, clinician-administered scale [20]; it is the most frequently used scale for the assessment of PD. The Japanese version of the PDSS was developed by Katagami [29].

In order to ensure that our results are comparable with those of previous studies of CBT, patients also completed additional self-report measures of PD severity: the Panic and Agoraphobia Scale (PAS), 9-item patient health questionnaire (PHQ-9), 7-item generalized anxiety disorder scale (GAD-7), and Brief Fear of Negative Evaluation Scale (BFNE). The Japanese versions of all of these measures have good reliability and validity.

The PAS [30] comprises 13 items that measure the severity of panic symptoms on a 5-point Likert-type scale. The Japanese version of the PAS was developed by Kaiya, Yoshida, and Kumano [31].

The PHQ-9 [32] contains nine items assessing severity of depression rated on a 4-point Likert-type scale. The Japanese version of the PHQ-9 was developed by Muramatsu et al. [33].

The GAD-7 [34] comprises seven items that measure the severity of generalized anxiety disorder on a 4-point Likert-type scale. The Japanese version of the GAD-7 was developed by Muramatsu [35].

The BFNE [36] contains 12 items that measure social fears on a 5-point Likert-type scale. BFNE is specifically intended to measure the social discomfort resulting from perceptions of being negatively evaluated by others, which is also relevant to PD. This was a short-form version adapted from the original 30-item scale [37]. The Japanese version of the BFNE was developed by Sasagawa et al. [38].

Patients also completed the 3-level version of EuroQol’s EQ-5D questionnaire. The EQ-5D [39] contains five items that assess quality of life on a 3-point Likert-type scale ranging from 1 (not severe) to 3 (severe). The Japanese version of the EQ-5D was developed by Tsuchiya et al. [40]. The EQ-5D is the most commonly used scale internationally for calculating QALYs. QALYs are often used in cost-utility analyses as the health outcome of choice; they are typically estimated via area-under-the-curve (AUC) analysis, which involves summing the areas of the distribution shapes for utility scores over the study period [41]. In the present study, QALYs were assessed using the EQ-5D index, an indicator of patient health status. This index is calculated by transforming the EQ-5D dimension scores into a single summary score ranging from 0 to 1 (1 = full health) by applying a formula created by the EuroQol Group [39]. Patients completed the questionnaires at home.

### Statistical analysis

All statistical tests were two-tailed, and an alpha level of 0.05 was employed. All data were analyzed using SPSS for Windows version 21 (SPSS Inc., Chicago, IL, USA). The outcomes of the CBT for PD were quantified as follows. First, regarding our primary outcome (PDSS scores), we analyzed changes between pre-CBT and the other two time points (mid-CBT and post-CBT) using repeated-measures, within-subjects ANOVAs. Furthermore, we established the following threshold for response and remission [20]: individuals were defined as “treatment responders” if they exhibited a 40 % or greater reduction in PDSS score over the course of treatment, while they were considered “in remission” if they had a score of 7 or less on the PDSS after the intervention [20]. We also calculated Cohen’s *d*, a measure of effect size, as the difference between the means divided by the pooled SD. According to Cohen [42], effect sizes are categorized as follows: small (0.20–0.49), medium (0.50–0.79), and large (0.80 and above).

To measure the cost-effectiveness of the CBT, we calculated QALYs at mid- and post-CBT using the AUC of changes in EQ-5D index from baseline [39]. Because of the lack of follow-up data, we estimated QALYs at 12 months after the start of CBT in the following two conditions: the worst condition, wherein the EQ-5D index had decreased to baseline at 12 months; and the best condition, wherein the EQ-5D index remained high at 12 months. Finally, to examine the other secondary outcomes, we compared the PAS, PHQ-9, GAD-7, and BFNE scores between pre-, mid- and post-CBT.

## Results

### Participant characteristics

All participating therapists adhered to the treatment protocol under supervision. Of the 17 subjects screened, 15 were eligible for participation and were recruited.

**Fig. 1:**
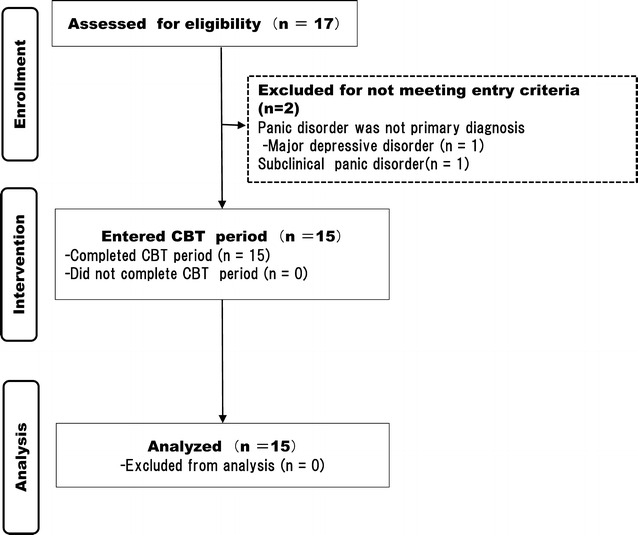
Participant flow diagram. *PDSS* Panic Disorder Severity Scale, *CBT* cognitive behavioral therapy

There were no dropouts over the course of the intervention. After enrolling in the study, no patients dropped out (Fig. 1). Table 1 shows the baseline demographic and clinical characteristics of the 15 patients. There were 13 women (80 %), and patients’ mean age was 38.6 years; 3 patients (20 %) were unemployed and 6 (40 %) were single, and their mean length of education was 12.7 years.

**Table 1:** Baseline demographic and clinical characteristics (N = 15)

Variable	Value
Female, n (%)	13 (80)
Age (years), mean (SD)	38.6 (9.6)
Comorbid agoraphobia, n (%) (M.I.N.I.)	13 (87)
Comorbid axis I diagnosis, n (%) (M.I.N.I.)	
No comorbid condition (PD only)	12 (80)
Major depression	1 (7)
Other anxiety disorder	3 (20)
Age of onset (years), mean (SD)	27.8 (9.5)
Duration of PD, years, mean (SD)	10.8 (9.5)
Employ status, n (%)	
Employed full-time	5 (33)
Full-time student	0 (0)
Part-time/homemaker	7 (47)
Unemployed	3 (20)
Marital status, n (%)	
Single	6 (40)
Married	8 (53)
Divorced	1 (7)
Educational background, n (%)	
Junior high school	0 (0)
High school	2 (13)
<3 years of college/university	8 (53)
≥3 years of college/university	5 (33)
Length of education (years), mean (SD)	12.7 (2.1)
Current medication, n (%)	
BZ	11 (73)
AD	9 (60)
Both BZ and AD	8 (53)
No medication	3 (20)

*PD* panic disorder, *BZ* benzodiazepine, *AD* antidepressant, *M.I.N.I.* Mini International Neuropsychiatric Interview

According to the Organisation for Economic Co-Operation and Development’s “Education At a Glance 2010,” the ratio of university graduates in the Japanese population ranges from 55.1 to 26.0 % among young (25–34 years old) and old- and middle-aged individuals (55–64 years old), respectively. Because the proportion of university graduates in this study was 53 %, which suggests that the sample was similar to the rate in the general population.

All participants met the DSM-5 diagnostic criteria for PD (mean duration of illness 10.8 years). Furthermore, 13 patients (87 %) also met the criteria for agoraphobia, 1 patient (7 %) for major depressive disorder, and 3 patients (20 %) for other anxiety disorders. Among the three patients with other anxiety disorders, two had generalized anxiety disorder, one patient had comorbid generalized anxiety disorder and social anxiety disorder. Nine patients (60 %) took antidepressants. Specifically, five patients took sertraline, one took escitalopram, one took paroxetine, one took both paroxetine and duloxetine, one took both sertraline and imipramine. Notably, all nine of these patients remained symptomatic despite adequate treatment with at least one SSRI at the maximum dose for at least 12 weeks; in other words, they exhibited intolerance to at least one SSRI [25]. There were no changes in pharmacotherapy during the CBT intervention.

### Primary outcome

**Fig. 2:**
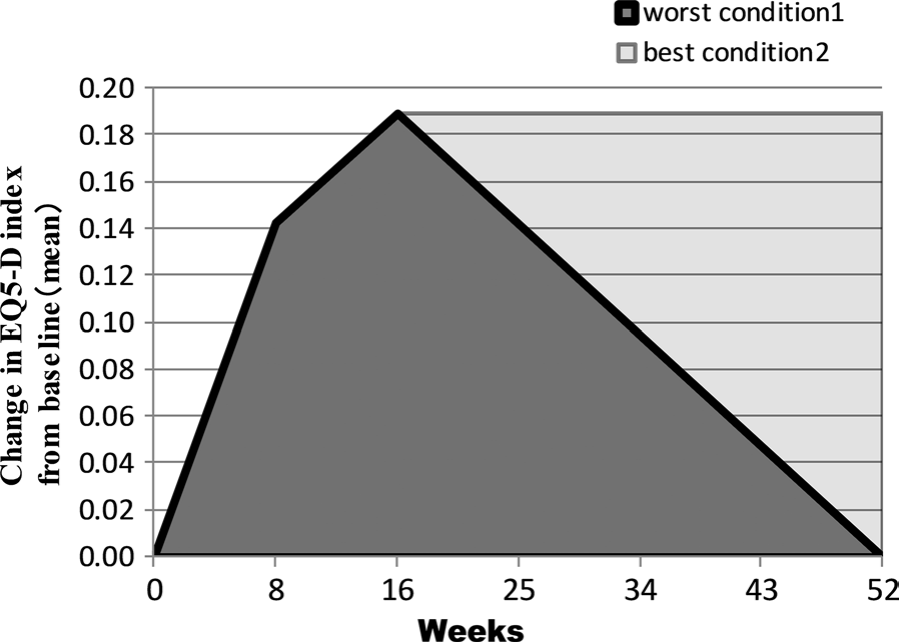
Estimated change in QALYs from baseline to 52 weeks (12 months). *QALY* quality-adjusted life year

Figure 2 and Table 2 show the outcome measures at each time point. The mean total PDSS score decreased from 12.1 at pre-CBT to 5.5 at post-CBT. A repeated-measures ANOVA revealed a significant main effect of time point on the PDSS total score, F (2, 42) = 12.39, p < 0.001 (see Fig. 2). Notably, 10 patients (66.7 %) met the criteria for remission of PD at post-CBT [20], and 10 patients (66.7 %) were judged to be responders [43]. PD remission was defined as having a score of seven or less on the PDSS, whereas a responder was defined as someone who showed a 40 % or greater reduction in PDSS score. In this study, patients overlapped between these groups.

**Table 2:** Outcome measures at each assessment point

	PDSS		PAS		PHQ-9		GAD-7		BFNE		EQ-5D index	
	Mean	SD	Mean	SD	Mean	SD	Mean	SD	Mean	SD	Mean	SD
Pre-CBT	12.1	4.0	23.5	5.8	8.0	3.2	8.7	5.1	42.7	12.4	0.665	0.2
Mid-CBT	7.5	3.3	15.3	3.6	5.4	2.5	5.1	3.6	34.3	12.1	0.823	0.1
Post-CBT	5.5	3.5	11.6	5.7	5.2	3.1	4.5	3.3	31.7	12.6	0.864	0.1
Pre-post CBT^a^	−6.6	4.3***	−11.9	6.6***	−2.8	3.6**	−4.2	3.6**	−10.9	9.2 (n.s.)	0.199	0.20**
Effect size	1.77		2.06		0.89		0.97		0.87		1.08	

*PDSS* Panic Disorder Severity Scale, *PAS* Panic and Agoraphobia Scale, *BFNE* Brief Fear of Negative Evaluation Scale, *PHQ-9* 9-item patient health questionnaire, *GAD-7* 7-item generalized anxiety disorder scale

*** *p* < 0.001, ** *p* < 0.01

^a^Mean changes from pre- to post-CBT time points

As shown in Table 3, the pre-to-post-CBT effect size (*d* = 1.77) was large, and provided comparable effectiveness to calculated for a previous study on individual CBT for PD [9].

**Table 3:** Comparison of effect sizes of CBT on Panic Disorder Severity Scale scores

Study group	CBT protocol	N	Mean per item		ES
			Pre	Post	
			(SD)	(SD)	
Present study	60 min	15	1.7	0.8	1.77
	16 weeks		0.6	0.5	
Barlow et al. [10]^a^	CBT	77	1.82	1.14	1.04
	12 weeks		(0.6)	(0.7)	
	Imipramine	83	1.88	1.05	1.23
	12 weeks		(0.6)	(0.8)	
	Placebo	24	1.88	1.52	0.46
	12 weeks		(0.5)	(0.9)	
	CBT+ imipramine	65	1.86	0.88	1.48
	12 weeks		(0.6)	(0.7)	
	CBT+ placebo	63	1.74	0.99	1.22
	12 weeks		(0.5)	(0.7)	

^a^Mean represents the average value of one item. The effect sizes reported are based on our calculations

### Secondary outcomes

#### PAS, PHQ-9, GAD-7, and BFNE

The mean total score of the PAS decreased from 23.5 at pre-CBT to 11.6 at post-CBT. We also noted significant improvements in the PHQ-9 and GAD-7 between pre- and post-CBT scores (*p* < 0.05). Although the BFNE scores did not significantly differ between the time points (see Table 2), they nevertheless showed large pre-to-post-CBT effect sizes (*d* = 0.85). The effect sizes for the PAS, GAD-7, and PHQ-9 score changes were also large, at 2.00, 0.95, and 0.86, respectively.

#### EQ-5D and QALYs

Table 4 shows the changes in each dimension score of the EQ-5D. Although all five dimension scores improved, only those of usual activities and pain/discomfort were significant.

**Table 4:** EQ-5D dimensions at each assessment point

	Mobility		Self-care		Usual activities		Pain/discomfort		Anxiety/depression		EQ-5D	
	Mean	SD	Mean	SD	Mean	SD	Mean	SD	Mean	SD	Mean	SD
Pre-CBT	1.2	0.6	1.1	0.3	1.7	0.6	1.9	0.7	1.9	0.7	0.665	0.2
Mid-CBT	1.3	0.5	1.0	0.0	1.3	0.5	1.5	0.5	1.3	0.5	0.823	0.1
Post-CBT	1.0	0.0	1.0	0.0	1.1	0.4	1.3	0.5	1.5	0.5	0.864	0.1
Pre-post CBT^a^	0.2		0.1		0.6	**	0.6	*	0.5	*	0.199	**
ES	0.47		0.00		1.18		0.99		0.66		1.08	

** *p* < 0.01, * *p* < 0.05

^a^Significantly different between pre- and post-CBT periods

The mean changes in the EQ-5D index from baseline were 0.143 at mid-CBT and 0.199 at post-CBT. According to the AUCs, the change in QALYs from baseline to post-CBT (i.e., 16 weeks) was estimated as 0.0364 QALYs. Under the worst condition—namely, that EQ-5D deteriorated to baseline at 12 months—the change in QALYs from baseline to 12 months was estimated as 0.102 QALYs. Under the best conditions—namely, that EQ-5D maintained a high level at 12 months—the change in QALYs from baseline was estimated as 0.178 QALYs. Therefore, between 0.102 and 0.178 QALYs were gained per 1 year.

Willingness-to-pay (WTP) values per QALY gained have been estimated in a past study as JPY 5 million (Japan), KWN 68 million (Republic of Korea), NT$ 2.1 million (Taiwan), 23,000 UK pounds (United Kingdom), AU$ 64,000 (Australia), and US$ 62,000 (United States; [44]). Using these values to convert the change in QALYs per 1 year into WTP values, we obtained values of JPY 543,000–889,000 (Japan) and US$ 6740–11,000 (United States). Because we provided patients 16 sessions of CBT, we estimated that patients would spend JPY 31,800–52,300 (Japan) and US$ 421–689 (US) per one session (50 min) of CBT. Incidentally, patients typically pay only around JPY 5000 per one session of CBT in the Japanese health insurance system at present.

## Discussion

This uncontrolled trial in Japan demonstrated that an individual CBT for PD improved PDSS scores, scores for various other measures of symptom severity, and QALYs. Regarding the primary outcome (the PDDS), in the acute phase after treatment, the change in PDSS score (effect size = 1.77) that we found was comparable to those that we calculated for a previous clinical trial conducted by Barlow et al. [10]. Specifically, for that trial, the effect sizes (Cohen’s *d*) for CBT, imipramine, placebo, CBT+ imipramine, and CBT+ placebo were 1.24, 1.48, 0.69, 1.72 and 1.41, respectively. Although our study was uncontrolled, these results appear promising.

The developed CBT for PD also appeared to improve the PAS score from 23.5 at pre-CBT to 11.6 at post-CBT (Cohen’s *d* = 2.06). King et al. [45] reported that 25 patients who received 16 sessions of CBT along with medication also showed a significant improvement in PAS, decreasing from 27.9 at pre-CBT to 18.6 at post-CBT (*p* = 0.012). Seo, Chow, Chung, Rho, and Chae [46] also reported that fourteen subjects who completed a group-based CBT showed an improvement in PAS from 24.86 (SD = 11.98) at pre-CBT to 14.8 (SD = 6.93) at post-CBT (*t* = 4.55, *p* = 0.001; Cohen’s *d* = 1.02). Considering these previous reports, our CBT appears to have high effectiveness in reducing panic symptoms according to both the PDSS and PAS. We also noted significant reductions in all other secondary outcomes—including depression (PHQ-9), generalized anxiety (GAD-7), and functional impairment (EQ-5D)—except for social anxiety (BFNE). One possible reason why our CBT for PD had little effect on improving social anxiety symptoms measured by the BFNE is that the SD of the BFNE score was too high because patients with PD had highly variable degrees of social anxiety symptoms.

We noted that individual CBT appears to be a feasible treatment for PD with major depressive disorder or other anxiety disorders in Japanese clinical settings. Most patients (80 %) in the current study (Table 1) were taking benzodiazepines or antidepressants, as Japanese public health insurance covers pharmacotherapy but not CBT for PD at this time. Heldt et al. [47] reported that CBT for pharmacotherapy-resistant patients appears to be effective in treating PD. In a future randomized controlled study on our CBT for PD, we intend to recruit pharmacotherapy-resistant patients to guide development of the next-step strategies in Japan.

Regarding the results for QALYs, we found that our CBT for PD resulted in somewhat higher gains for QALYs compared with previous studies on other disorders. For instance, Grochtdreis et al. [48] reported that collaborative care for the treatment of depressive disorders in primary care offered a mean incremental gain of 0.02 QALYs over 12 months, compared with usual care, in their systematic review of 19 cost-effectiveness analyses. McCrone et al. [49] reported that CBT for chronic fatigue syndrome had an incremental gain of 0.05 QALYs at 12 months, compared with specialist medical care alone, after controlling for baseline utility. Mukuria et al. [50] reported that an “improving access to psychological therapies” service (covering effective psychological therapies for common mental health problems, such as depression and anxiety) in the United Kingdom provided an incremental gain of 0.014 QALYs, while a cost-benefit analysis of psychological therapies including CBT undertaken by Layard et al. [51] estimated that the QALYs gained would be 0.11. Overall, our results suggest the CBT for PD developed in the present study might be highly cost-effective.

## Limitations

Overall, although our present study provides highly valuable information, it does have some limitations, including its small sample size and lack of a control group, controlled pharmacotherapy, and long-term follow-up data. Without a placebo control group, it remains unknown whether the observed improvements in PD severity are merely the result of the natural course of PD or a result of the intervention. When judging the effectiveness of a treatment for PD, it is important to consider the placebo effects noted in previous reports [10, 52–54]. Thus, future studies should employ psychological placebo conditions to control for nonspecific factors, such as positive outcome expectancy and self-efficacy enhancements related to starting to manage their problems. In the near future, we intend to conduct a randomized controlled trial that includes long-term follow-up to provide greater insight into this CBT for PD in routine Japanese practice, based on the results of the current study. In this study, 80 % of our patients were on medication and thus we cannot conclude whether the CBT for PD will be effective for patients not receiving pharmacotherapy. It would thus be necessary to investigate CBT during drug-free periods. In the near future, a three-armed randomized controlled trial comparing pill placebo (as the control group), CBT patients on antidepressants, and CBT patients who are drug-free should be designed and performed.

## Conclusions

Despite the limitations, our results suggest that CBT is a feasible treatment that is potentially cost-effective for treating PD in Japanese clinical settings. Further randomized controlled trials that address the limitations of this study are required.

## Abbreviations

CBT: cognitive behavioral therapy
PD: panic disorder
QALY: quality-adjusted life year
WTP: willingness-to-pay
PDSS: Panic Disorder Severity Scale
PAS: Panic and Agoraphobia Scale
BFNE: Brief Fear of Negative Evaluation Scale
PHQ-9: 9-item patient health questionnaire
GAD-7: 7-item generalized anxiety disorder scale
BZ: benzodiazepine
AD: antidepressant
M.I.N.I.: Mini International Neuropsychiatric Interview
