# Erratum to: A feasibility study of the clinical effectiveness and cost-effectiveness of individual cognitive behavioral therapy for panic disorder in a Japanese clinical setting: an uncontrolled pilot study

**DOI:** 10.1186/s13104-017-2398-y

**Published:** 2017-02-06

**Authors:** Yoichi Seki, Shinobu Nagata, Takayuki Shibuya, Naoki Yoshinaga, Mizue Yokoo, Hanae Ibuki, Noriko Minamitani, Muga Kusunoki, Yasushi Inada, Nobuko Kawasoe, Soichiro Adachi, Kensuke Yoshimura, Michiko Nakazato, Masaomi Iyo, Akiko Nakagawa, Eiji Shimizu

**Affiliations:** 1 United Graduate School of Child Development, Osaka University, Kanazawa University, Hamamatsu University School of Medicine, Chiba University and University of Fukui, Suita, Japan; 2 Research Center for Child Mental Development, Chiba University Graduate School of Medicine, 1-8-1 Inohana, Chuo-ku, Chiba-shi, Chiba, 260-8670 Japan; 3 Department of Cognitive Behavioral Physiology, Chiba University Graduate School of Medicine, Chiba, Japan; 4 Organization for Promotion of Tenure Track, University of Miyazaki, Miyazaki, Japan; 5 Inada Clinic, Osaka, Japan; 6 Clinic Adachi, Gifu, Japan; 7 Department of Psychiatry, Graduate School of Medicine, Chiba University, Chiba, Japan

## Erratum to: BMC Res Notes (2016) 9:458 DOI 10.1186/s13104-016-2262-5

After publication of the original article [1], it came to the authors’ attention that there were typing errors in the data presented in Tables 2 and 4. The values in the EQ-5D index column for both tables were mistyped, and the correct versions (Tables 2 and 4) of both tables are published in this erratum.

**Table 2:** Outcome measures at each assessment point

	PDSS		PAS		PHQ-9		GAD-7		BFNE		EQ-5D index	
	Mean	SD	Mean	SD	Mean	SD	Mean	SD	Mean	SD	Mean	SD
Pre-CBT	12.1	4.0	23.5	5.8	8.0	3.2	8.7	5.1	42.7	12.4	0.665	0.2
Mid-CBT	7.5	3.3	15.3	3.6	5.4	2.5	5.1	3.6	34.3	12.1	0.807	0.2
Post-CBT	5.5	3.5	11.6	5.7	5.2	3.1	4.5	3.3	31.7	12.6	0.854	0.1
Pre-post CBT^a^	−6.6	4.3***	−11.9	6.6***	−2.8	3.6**	−4.2	3.6**	−10.9	9.2 (ns)	0.189	0.20**
Effect size	1.77		2.06		0.89		0.97		0.87		0.88	

*PDSS* Panic Disorder Severity Scale, *PAS* Panic and Agoraphobia Scale, *BFNE* Brief Fear of Negative Evaluation Scale, *PHQ-9* 9-item patient health questionnaire, *GAD-7* 7-item generalized anxiety disorder scale

*** *p* < 0.001, ** *p* < 0.01

^a^Mean changes from pre- to post-CBT time points

**Table 4:** EQ-5D dimensions at each assessment point

	Mobility		Self-care		Usual activities		Pain/discomfort		Anxiety/depression		EQ-5D	
	Mean	SD	Mean	SD	Mean	SD	Mean	SD	Mean	SD	Mean	SD
Pre-CBT	1.2	0.6	1.1	0.3	1.7	0.6	1.9	0.7	1.9	0.7	0.665	0.2
Mid-CBT	1.3	0.5	1.0	0.0	1.3	0.5	1.5	0.5	1.3	0.5	0.807	0.2
Post-CBT	1.0	0.0	1.0	0.0	1.1	0.4	1.3	0.5	1.5	0.5	0.854	0.1
Pre-post CBT^a^	0.2		0.1		0.6	**	0.6	*	0.5	*	0.189	**
ES	0.47		0.00		1.18		0.99		0.66		0.88	

** *p* < 0.01, * *p* < 0.05

^a^Significantly different between pre- and post-CBT periods

In addition, there were errors in the EQ‑5D and QALYs sub-section of the Results.

In the second paragraph, the following sentence has been amended to change the post-CBT value from 0.199 to 0.189: “The mean changes in the EQ-5D index from baseline were 0.143 at mid-CBT and 0.189 at post-CBT.”

In the same paragraph, the final sentence has been amended to correct the change in QALYs from 0.178 to 0.167: “Under the best conditions—namely, that EQ-5D maintained a high level at 12 months—the change in QALYs from baseline was estimated as 0.167 QALYs. Therefore, between 0.102 and 0.167 QALYs were gained per 1 year.”

Finally, in the last paragraph of the sub-section, the JPY and US$ values have been corrected: “Using these values to convert the change in QALYs per 1 year into WTP values, we obtained values of JPY 510,000–835,000 (Japan) and US$ 6320–10,350 (United States). Because we provided patients 16 sessions of CBT, we estimated that patients would spend JPY 31,800–52,100 (Japan) and US$ 395–647 (US) per one session (50 min) of CBT.”
